# Disentangling the Amyloid Pathways: A Mechanistic Approach to Etiology

**DOI:** 10.3389/fnins.2020.00256

**Published:** 2020-04-21

**Authors:** Maja Malmberg, Tarja Malm, Oskar Gustafsson, Andrea Sturchio, Caroline Graff, Alberto J. Espay, Anthony P. Wright, Samir El Andaloussi, Anders Lindén, Kariem Ezzat

**Affiliations:** ^1^Section of Virology, Department of Biomedical Sciences and Veterinary Public Health, Swedish University of Agricultural Sciences, Uppsala, Sweden; ^2^SLU Global Bioinformatics Centre, Department of Animal Breeding and Genetics, Swedish University of Agricultural Sciences, Uppsala, Sweden; ^3^A.I. Virtanen Institute for Molecular Sciences, University of Eastern Finland, Kuopio, Finland; ^4^Department of Laboratory Medicine, Clinical Research Center, Karolinska Institutet, Stockholm, Sweden; ^5^Department of Neurology and Rehabilitation Medicine, James J and Joan A Gardner Center for Parkinson Disease and Movement Disorders, University of Cincinnati, Cincinnati, OH, United States; ^6^Department of Neurobiology, Care Sciences and Society, Karolinska Institutet, Solna, Sweden; ^7^Unit for Hereditary Dementias, Theme Aging, Karolinska University Hospital, Solna, Sweden; ^8^Department of Physiology, Anatomy and Genetics, University of Oxford, Oxford, United Kingdom; ^9^Unit for Lung and Airway Research, Institute of Environmental Medicine, Karolinska Institutet, Stockholm, Sweden; ^10^Department of Respiratory Medicine and Allergy, Karolinska University Hospital, Stockholm, Sweden

**Keywords:** amyloid, nucleation, Alzheiemr’s, Parkinson’s, virus, prion, protein-only hypothesis

## Abstract

Amyloids are fibrillar protein aggregates associated with diseases such as Alzheimer’s disease (AD), Parkinson’s disease (PD), type II diabetes and Creutzfeldt–Jakob disease. The process of amyloid polymerization involves three pathological protein transformations; from natively folded conformation to the cross-β conformation, from biophysically soluble to insoluble, and from biologically functional to non-functional. While amyloids share a similar cross-β conformation, the biophysical transformation can either take place spontaneously via a homogeneous nucleation mechanism (HON) or catalytically on an exogenous surface via a heterogeneous nucleation mechanism (HEN). Here, we postulate that the different nucleation pathways can serve as a mechanistic basis for an etiological classification of amyloidopathies, where hereditary forms generally follow the HON pathway, while sporadic forms follow seed-induced (prions) or surface-induced (including microbially induced) HEN pathways. Critically, the conformational and biophysical amyloid transformation results in loss-of-function (LOF) of the original natively folded and soluble protein. This LOF can, at least initially, be the mechanism of amyloid toxicity even before amyloid accumulation reaches toxic levels. By highlighting the important role of non-protein species in amyloid formation and LOF mechanisms of toxicity, we propose a generalized mechanistic framework that could help better understand the diverse etiology of amyloid diseases and offer new opportunities for therapeutic interventions, including replacement therapies.

## Introduction

The term amyloid refers to a particular conformational state of proteins where they transform from being soluble and natively folded into insoluble aggregates of fibrillar nature. More than 35 peptides and proteins are known to form amyloids in different human diseases (Chiti and Dobson, 2017). Nearly all the proteins that form amyloids have biological functions in their normal, natively folded state. Some proteins such as antibodies, lipoproteins and serum amyloid (SAA) lead to systemic amyloidosis including light-chain amyloidosis, Apo-AI amyloidosis and AA amyloidosis, respectively (Chiti and Dobson, 2017). Other proteins accumulate in specific organs leading to localized amyloid pathology. These amyloidopathies include thyroid medullary carcinoma, pulmonary alveolar proteinosis and atrial amyloidosis resulting from the amyloid accumulation of calcitonin, surfactant protein C and atrial natriuretic factor, respectively (Chiti and Dobson, 2017). Localized amyloidopathies also include type II diabetes and neurodegenerative diseases such as Alzheimer’s disease (AD) and Parkinson’s disease (PD). Type II diabetes is characterized by the amyloid accumulation of the peptide hormone islet amyloid polypeptide (IAPP), while AD and PD are characterized by the accumulation of the amyloid beta (Aβ) and alpha synuclein (α-syn) peptides, respectively (Eisenberg and Jucker, 2012). Moreover, AD and other neurodegenerative diseases such as frontal temporal dementia with Parkinsonism and Pick’s disease involve amyloid aggregates of the microtubule-associated protein tau (Gendron, 2009). In addition, the amyloid aggregation of the infamous tumor suppressor transcription factor p53 is involved in many cancers (Navalkar et al., 2019). While some amyloids were shown to have beneficial biological function, for example acting as storage for peptide hormones in secretory granules (Jackson and Hewitt, 2017), the vast majority of amyloids are pathological. This explains the existence of several biological protective mechanisms that ensure that proteins are correctly folded such as the presence of chaperones, or degraded when incorrectly folded via processes such as autophagy, ubiquitin–proteasome mediated degradation, and the unfolded protein response (Knowles et al., 2014). Moreover, specific sequence patterns that tend to easily form amyloids, such as alternating hydrophilic-hydrophobic stretches, appear to have been selected against during evolution (Broome and Hecht, 2000; Shoulders and Raines, 2009).

## Etiology of Amyloidopathies

A small proportion of amyloidopathies is of genetic hereditary origin; however, the majority of amyloid diseases are sporadic (Chiti and Dobson, 2017). Hereditary forms of amyloidopathies are caused by mutations in the genes encoding the amyloidogenic proteins, either via gene duplication or mutations that facilitate protein aggregation leading to early onset of the disease (more details below). For sporadic forms, a small subset of amyloidopathies, termed transmissible spongiform encephalopathies (TSEs), are caused by infectious protein particles called prions (Cobb and Surewicz, 2009). Prions transfer from one organism to another inducing neurodegeneration in the recipient host in diseases such as Creutzfeldt–Jakob disease and Kuru. For the vast majority of other sporadic forms, the causes remain unclear. However, several environmental factors are known to increase disease risk, including infections (Itzhaki et al., 2016), lipid dysregulation (Mesa-Herrera et al., 2019), pollution (Kilian and Kitazawa, 2018), and traumatic brain injury (Johnson et al., 2010).

## Amyloid Structure

**FIGURE 1:**
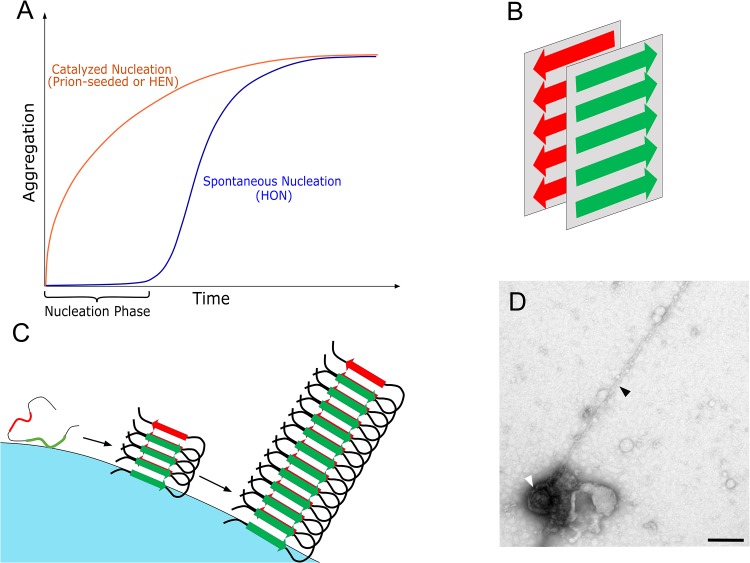
**(A)** A schematic representation of the kinetics of amyloid aggregation with the rate-limiting nucleation phase that can be bypassed either by adding a preformed seed (prion) or by surface catalysis via HEN. **(B)** A schematic representation of the cross-β conformation, which is the core conformation of amyloids where two β-sheets are stacked opposite to each other forming the protofilament with the characteristic elongated amyloid morphology. While the cross-β conformation remains constant, variable β-sheet orientations or protofilament associations lead to different amyloid polymorphs. **(C)** A schematic representation of HEN where an exogenous surface catalyze amyloid nucleation via binding, concentrating, and inducing conformational changes in the bound peptides/proteins, which facilitate amyloid transformation. **(D)** A negatively stained transmission electron microscopy picture demonstrating direct interaction between the surface of a virus (HSV-1) and a growing amyloid protofilament of Aβ 1–42 peptide (unpublished image from the viral catalyzed nucleation experiments performed in the study of Ezzat et al. (2019), where HSV-1 was incubated with 50 μM Aβ 1–42 for 100 min. at 37°C). In the same publication, we demonstrated that HSV-1 accelerated amyloid aggregation *in vitro* and *in vivo*. The viral particle is indicated by a white arrow and the protofilament with a black arrow, bar = 200 nm.

The term amyloid describes a unique class of protein conformation, where proteins adopt elongated fibrillar morphology. While this is a characteristic feature of pathological protein aggregates, it has been demonstrated that even normal non-pathogenic proteins can be forced to adopt the amyloid conformation under certain denaturing conditions (Fändrich et al., 2001). This led to the “generic hypothesis” suggesting that amyloid formation originates from the fundamental properties of proteins, based on the ability of backbone groups to form hydrogen bonds and the ability of side-chain groups to interact via hydrophobic and van der Waals interactions (Auer et al., 2007). To obtain their characteristic morphology, amyloids share a similar core cross-β conformation (Eisenberg and Sawaya, 2017). In this conformation, the protein molecules are arranged in the form of two oppositely stacked β-sheets, excluding the water molecules in-between and interdigitating their side chains forming a dry steric zipper (Eisenberg and Jucker, 2012). Such an elongated cross-β spine constitutes the basic amyloid fibrillar subunit, the protofilament (Figure 1). Apart from the fixed cross-β conformation, protein stacking in the spine can come in a variety of forms. For example, the cross-β architecture can consist of one folded molecule or two separate molecules and the β-sheets can stack in parallel, anti-parallel, face-to-face or face-to-back orientations (Eisenberg and Sawaya, 2017). Moreover, the protofilament length varies depending on the number strands forming each sheet. Once protofilaments are formed, they can associate in a variety of ways leading to different superstructural polymorphs. These polymorphs include flat fibrillar structures with varied number of horizontally stacked protofilaments, which can evolve to amyloid crystals, or different twisted ribbon structures (of single or multiple intertwined protofilaments), and these can further evolve into nanotubes (Mezzenga and Adamcik, 2018). Such superstructural polymorphism depends on many factors including the protein side chain arrangements, the nucleation mechanism (see below) and environmental conditions such as pH, temperature and ion concentration (Morel et al., 2010; Tycko and Wickner, 2013). In addition, this biochemical structural transformation is accompanied by a biophysical phase transformation that is dominated by nucleation-growth kinetics as described below.

## Amyloid Nucleation Mechanisms

Phase transformation is the process involving transitioning from one state of matter to another, such as liquid to solid or gas to liquid or gas to solid transformations. These transformations are very common in nature, including phenomena such as crystallization and amyloid aggregation (liquid to solid transformation), rain precipitation (gas to liquid transformation) and planet formation (gas to solid transformation) (Karthika et al., 2016; Ros et al., 2019). That is why both the thermodynamics and kinetics of phase transformation have been widely studied. Thermodynamically, the process involves the transition from a less stable (higher free energy) to a more stable (lower free energy) phase under specified conditions (Mezzenga and Adamcik, 2018). Kinetically, the mechanism of phase transformation involves two steps that are well described by the classical nucleation theory (Karthika et al., 2016). Initially, a rate-limiting nucleation step takes place, where an energy barrier needs to be overcome to create the initial molecular assembly (nucleus) of the new phase. Once the nucleus is formed, this is followed by a growth step where the system rapidly transforms into the new phase. The nucleation-growth mechanism accurately describes the kinetics of amyloid formation as studied experimentally, with the distinctive sigmoidal kinetics curve involving a nucleation lag phase followed by a rapid growth or elongation phase (Knowles et al., 2014; Figure 1).

While nucleus formation is necessary, it is both thermodynamically unfavorable and rare, as it depends on the unlikely event of the spontaneous formation of a stable nucleus of the new phase within the bulk of the transforming phase (Vekilov, 2010; Eisenberg and Jucker, 2012). In the case of amyloid aggregation, this involves the spontaneous conformational change of the protein followed by spontaneous association of protein molecules into cross-β sheet rich nucleus. This pathway to nucleation is called homogenous nucleation (HON) (Buell, 2017), and with all the protective mechanisms that are in place to maintain proper protein folding (see above), it is not surprising that proteins do not normally reach the amyloid state. In contrast to the slow and rare HON pathway, catalyzed nucleation pathways exist that are faster and more common. One catalytic pathway is called seeding, where adding a preformed nucleus (seed/prion) enables the system to completely bypass the nucleation step and move directly to the growth or elongation step (Eisenberg and Jucker, 2012). The other important catalytic pathway is the heterogeneous nucleation mechanism (HEN), where an exogenous surface catalyzes the nucleation process (Buell, 2017; Srivastava et al., 2019). In HEN, the surface lowers the energy barrier to nucleation and acts as a scaffold that facilitates nucleus formation via binding, concentrating and enabling conformational changes in the bound proteins (Auer et al., 2009; John et al., 2018; Figure 1). Like seeding, HEN usually eradicates the lag phase completely from the kinetics. As an efficient nucleation mechanism, the polymerization of functional protein filaments such as actin or tubulin is dependent on HEN mechanisms, that are tightly controlled via sophisticated nucleator protein complexes such as γ-tubulin ring complex (Kollman et al., 2011) and the actin-related protein 2/3 complex (Campellone and Welch, 2010).

In the case of amyloids, many biological and non-biological surfaces have been shown to be capable of inducing amyloid aggregation via HEN. This includes microbial surfaces (Bantle et al., 2019; Ezzat et al., 2019), lipid vesicles (Habchi et al., 2018), and nanoparticles (Linse et al., 2007). Additionally, polymer surfaces such as glycosaminoglycans (GAGs) (Iannuzzi et al., 2015) and nucleic acids (Liu and Zhang, 2011) have been shown to accelerate amyloid aggregation. Moreover, the growing fibril surface itself can serve as a site for HEN, in a phenomenon termed secondary nucleation (Tö et al., 2018). Furthermore, the concentration of proteins in intracellular droplets that form via a liquid-liquid phase separation (LLPS) process can sometimes lead to amyloid formation (Brangwynne, 2017). Very recently, Yuan et al. (2019) have demonstrated that the interfaces created by LLPS can act as sites of HEN for amyloids (Yuan et al., 2019). While a variety of surfaces were shown to induce amyloid aggregation via HEN, the exact properties of a particular surface that mediate HEN of amyloids remain poorly understood. However, the main differences between HEN in normal protein polymerization and amyloid aggregation are the lack of controlled nucleation via nucleator complexes. Additionally, in normal processes, protein subunits assemble in their native rather than unfolded cross-β conformation.

## A Mechanistic Approach to Etiology

Amyloid aggregation is a process of pathological protein transformation at three levels, a biochemical conformational transformation, a biophysical phase transformation and a biological functional transformation. At the biochemical structural level, amyloids share a similar cross-β conformation across different pathologies and different polymorphs. At the biophysical level however, there are distinct nucleation-dependent pathways to amyloid formation that are well-defined in thermodynamic and kinetic terms. The nucleation barrier is what separates the soluble and insoluble states of a protein; and thus, the pathways to nucleation are the decisive mechanisms in the biophysical transformation process. The nucleation barrier dictates whether a protein would spontaneously form an amyloid via HON or whether it requires a catalytic event, which can be a preformed seed/prion or an exogenous surface (HEN). In addition, together with other environmental factors such as pH and ion concentration, it affects the final polymorphic superstructure (Srivastava et al., 2019).

**FIGURE 2:**
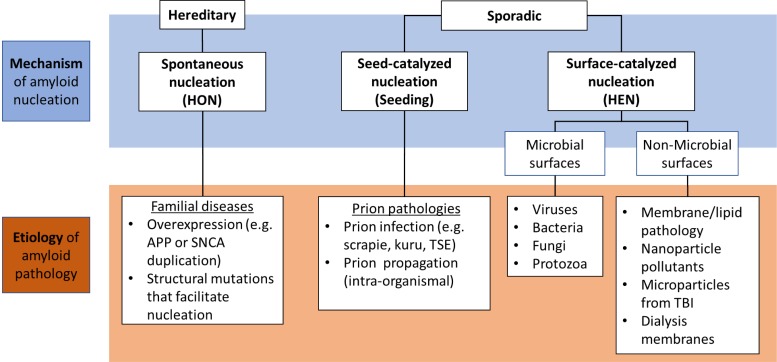
Classification of different amyloid etiologies in relation to the nucleation mechanisms where hereditary forms are usually caused by genetic mutations that facilitate HON, while sporadic forms are mainly catalyzed via prions or pathological nucleating surfaces (PNSs) which can be of microbial or non-microbial origin. APP, amyloid precursor protein; SNCA, α-synuclein gene; TSE, transmissible spongiform encephalopathy; TBI, traumatic brain injury.

Here, we propose that different nucleation pathways could also serve as the mechanistic basis for an etiological classification of amyloid pathologies (Figure 2). In this framework, HON is facilitated by mutations that render the protein more prone for spontaneous self-assembly; and hence, is expected to be the dominant mechanism in hereditary amyloidopathies. This includes structural mutations that facilitate spontaneous nucleus formation (Chiti et al., 2002; Kim and Hecht, 2008) or gene duplication and/or triplication, where the increased concentration of the protein would lead to lowering of the nucleation barrier increasing the probability of spontaneous nucleus formation (Auer et al., 2007). Such mutations include amyloid precursor protein (APP) gene duplication in Down syndrome and synuclein gene (SNCA) duplication in familial PD, both leading to early AD and PD pathologies, respectively (Konno et al., 2016; Lott and Head, 2019).

However, in the normal state in absence of mutations, the protein retains its native structure protected from aggregation by the nucleation barrier together with the other biological mechanisms that prevent proteins from going down the amyloid pathway (such as the chaperone and proteasome machinery). In this case, pathogenic catalytic mechanisms are required for the pathological transformation. This can take place via seeding by a preformed amyloid fragment (seed/prion) whose source can be the same organism due to amyloid fragmentation leading to prion propagation (intra-organismal), or a different organism resulting from a prion infection (Jucker and Walker, 2013). Alternatively, nucleation can be induced by aberrant surfaces that catalyze HEN. Such pathological nucleating surfaces (PNSs) can either be of endogenous origin due to a membrane or lipid pathology, or from an exogenous source such as microbes or nanoparticulate pollutants. These HEN catalysts can be responsible for the non-hereditary sporadic disease forms.

**BOX 1. In further support for the importance of LOF diseases mechanisms, in many disease models of amyloid diseases, knocking out/down the pathogenic protein leads to disease phenotypes in absence of plaques or aggregates.**

• α-synuclein (Abeliovich et al., 2000; Chandra et al., 2005; Gorbatyuk et al., 2010; Greten-Harrison et al., 2010; Collier et al., 2016).
• Amyloid Precursor Protein (APP) (Senechal et al., 2007; Tyan et al., 2012; Wang et al., 2012; Liu et al., 2019; Martinsson et al., 2019; Southam et al., 2019; Truong et al., 2019).
• TDP-43 (Kabashi et al., 2009; Yang et al., 2014; Prpar Mihevc et al., 2016).
• Tau (Kimura et al., 2014; Lei et al., 2014; Biundo et al., 2018; Velazquez et al., 2018).
• Superoxide dismutase 1 (SOD-1) (Saccon et al., 2013)^∗^.
• PrP (Sakaguchi et al., 1996; Criado et al., 2005; Bremer et al., 2010; Küffer et al., 2016).
• IAPP (Gebre-Medhin et al., 1998; Mulder et al., 2000).
• P53 (Donehower et al., 1992; Ano Bom et al., 2012; Lasagna-Reeves et al., 2013; Ghosh et al., 2017).

^∗^A comprehensive review of the evidence of LOF toxicity of SOD-1 from animal models and clinical data.

While non-protein factors have usually been considered “co-factors” to a “protein-only” driven process based on prion seeding (Soto, 2011), we emphasize that surfaces are independent causal factors as they are able to induce amyloid aggregation in the absence protein seeds/prions via the distinct and separate pathway of HEN. Lipid vesicles (Habchi et al., 2018), nanoparticles (Linse et al., 2007) and viruses (Ezzat et al., 2019) have been shown to induce amyloid aggregation via HEN in absence of preformed seeds. Additionally, being thermodynamically more favorable, HEN is more likely to be the prevalent pathway of amyloid aggregation *in-vivo* in the absence of genetic mutations that can facilitate HON. In this regard, microbes such as viruses and bacteria, which are capable of invading and reproducing in tissues, can be potent mediators of HEN in sporadic amyloidopathies. We have recently shown that viruses such as respiratory syncytial virus (RSV) and herpes simplex virus type 1 (HSV-1) are able to induce amyloid formation by catalyzing HEN of IAPP and Aβ, respectively (Ezzat et al., 2019). *In vivo*, HSV-1 intracranial infection in an AD animal model resulted in amyloid accumulation within 48 h post-infection (Ezzat et al., 2019). Similar observations were demonstrated for other pathogens such as bacteria and fungi (Kumar et al., 2016; Dominy et al., 2019). This shows that microbes are potent HEN inducers of amyloid aggregation. On the other hand, PNSs may arise from endogenous sources. These can be the result of lipid dysregulation involving lipoproteins such as ApoE ε4, which is a known genetic risk factor for AD (Potter and Wisniewski, 2012), or membrane components such as cholesterol, gangliosides and GAGs (Iannuzzi et al., 2015; Penke et al., 2018). Furthermore, membrane fragment microparticles from brain injury (Zhao et al., 2017) can potentially act as catalytic surfaces for HEN mediated amyloid aggregation in traumatic brain injury. Moreover, as has been reported for the amyloid aggregation of insulin (Nayak et al., 2008), synthetic membranes can act as sites for HEN mediated aggregation of some plasma proteins such as β2 microglobulin in dialysis-related amyloidosis (Scarpioni et al., 2016).

It can also be postulated that in some cases HON and HEN mechanisms can overlap, where mutations that would facilitate spontaneous amyloid aggregation via HON can also render the protein more vulnerable for surface-catalyzed amyloid transformation via HEN. Furthermore, HEN mechanisms could lead to distinctive amyloid superstructural polymorphs based on the properties of the catalyzing surface. Virus-induced amyloid aggregation, for example, can be expected to result in particularly deformed polymorphs due to HEN occurring on an acutely curved nanosurface. Crystalline deformation has been demonstrated before when crystallization takes place on a curved surface (Meng et al., 2014; Gómez et al., 2015). In the case of amyloids, horizontal stacking of protofilaments will be limited by the surface curvature. This, together with the possible existence of multiple nucleation sites on the same viral particle would lead to distinct polymorphic features that can act as histopathological hallmarks for viral-induced amyloidopathies, and can help trace back the etiology. Moreover, the conformational and phase transformations would result in pathogenic functional transformations that are described in the section “Gain or Loss of Function?”

## Gain or Loss of Function?

From a functional point-of-view, it has been difficult to correlate the pathogenicity of amyloids with particular structural features (Eisenberg and Jucker, 2012; Collinge, 2016). Here we postulate that while the gain-of-function (GOF) toxicity becomes more likely with increased amyloid accumulation in a tissue (especially in systemic forms of amyloidosis), a loss-of-function (LOF) toxicity likely constitutes the initial cytotoxic mechanism. Nearly all amyloid-forming proteins have known functions in their native folded state. Since any protein needs to adopt an appropriate conformation in order to perform its function, protein unfolding into the cross-β conformation accompanied by phase transformation into solid fibrils generally abolishes the native function of the protein. Proteins such as lipoproteins, antibodies and IAPP (also called amylin) are not able to perform their homeostatic, immunological, or hormonal functions in their pathological amyloid forms in Apo-AI amyloidosis, light-chain amyloidosis and diabetes, respectively (Hieronymus and Griffin, 2015; Muchtar et al., 2016; Lu et al., 2017). The same applies to Aβ, prion protein (PrP) and α-syn, which are the most studied in the context of neurodegenerative disorders. Soluble Aβ was shown to be important for synaptic plasticity and memory (Parihar and Brewer, 2010), soluble PrP on the other hand is involved in myelin maintenance and cellular proliferation processes (Castle and Gill, 2017), while α-syn is important for the regulation of neurotransmission and response to cellular stress (Benskey et al., 2016). Thus, it is expected that these original functions will be lost even before amyloid accumulation reaches substantial toxic GOF levels, and that LOF can at least initially be the neurodegenerative mechanism, as has been suggested previously (Saccon et al., 2013; Vanden Broeck et al., 2014; Benskey et al., 2016; Kepp, 2016).

The importance of LOF is further supported by the fact that in amyloid disease models, knocking out/down the protein results in disease phenotypes in the absence of the amyloidogenic protein and its aggregated forms (Box 1). Moreover, in AD for example, it has been repeatedly demonstrated that there is not always a correlation between the plaque load and disease severity. This has been shown in animal models (Hsia et al., 1999) and in healthy subjects with significant plaque load but without significant cognitive impairment (Aizenstein et al., 2008). One way to explain this paradox within a GOF framework has been to postulate that toxicity comes from a not-very-well-defined species called the amyloid oligomers, and not from the plaques (Selkoe, 2002). However, an additional explanation can be the fact that proteins that lose their native conformation will instantly lose their function even if they do not become particularly more toxic, and that such LOF contributes to neuronal degeneration. This is further illustrated by neuronal phenotypes in knockout animal models of several different amyloid pathologies (Box 1). Moreover, in many amyloidopathies, the CSF levels of the soluble peptides/proteins that eventually form amyloid aggregates decrease rather than increase (Kang, 2016; Hu et al., 2017). In AD and PD, the decrease in CSF levels of soluble Aβ (Fagan et al., 2009; Bateman et al., 2012; Buchhave et al., 2012) and α-syn (Tokuda et al., 2006; Wang et al., 2015; Mollenhauer et al., 2019; Parnetti et al., 2019) is among the early biochemical markers of the disease. A similar downward trend is observed for the CSF levels of soluble PrP in CJD (Meyne et al., 2009; Dorey et al., 2015). This indicates that the soluble to insoluble transformation, which would be accompanied by decreased levels and consequential LOF of the soluble fraction, might be among the earliest pathological changes. Notably, lower levels of soluble Aβ and α-syn are present even in patients with APP gene duplication in Down syndrome (Tapiola et al., 2001; Portelius et al., 2014) and SNCA gene duplication in familial PD (Kasuga et al., 2010). This suggests that pathological overexpression, which would facilitate HON by lowering the nucleation barrier, can also lead to decreased levels and LOF of the soluble proteins, which will be sequestered in the amyloid form. Taken together with data from knockout and knock down studies and the poor correlation between the amyloid plaque load and disease severity, these different lines of evidence strongly suggest an critical role of LOF mechanisms in the pathophysiology of amyloid diseases.

Furthermore, it has been shown that amyloid fragments (seeds/prions) can propagate pathology from one region to another within the same organism in diseases such as AD and PD (Brundin et al., 2010; Steiner et al., 2011). In this case, seeds/prions will induce LOF phase transformation when they encounter a new protein pool. The same can be true for HEN induced by microbes, where the ability of a pathogen to infect a particular area would lead to LOF amyloid transformation in that area. This may explain why in some neurodegenerative diseases the spread of the pathology follows the anatomical connections, which are the same routes for both prion and microbial propagation (Hawkes et al., 2007; Brettschneider et al., 2015). The LOF framework might also explain the failure of therapeutic approaches aiming only to reduce the amyloid forming proteins and open new directions in treatment that include restoring protein homeostasis via replacement therapy with functional, non-aggregating forms of the protein (Mockett et al., 2017). Indeed, synthetic IAPP (amylin) analogs such as pramlintide are clinically used as replacement therapy in diabetes (Hieronymus and Griffin, 2015). Furthermore, overexpression of soluble amyloid precursor protein alpha (APPsα) has been shown to restore synaptic plasticity, and rescue spatial memory in an AD mouse model with preexisting pathology and amyloidosis (Fol et al., 2016). This demonstrates that replacement therapy within a LOF framework is a promising approach; one that can be extended to other amyloidopathies.

## Phase Transformation or Replication?

The amyloid aggregation phenomenon, especially in the context of prions, is sometimes referred to as a process of protein “self-replication” that is dominated by a “protein-only” species leading to different prion “strains” that possess different pathogenic potentials (Collinge, 2016). Here, we argue that the phenomenon of amyloid aggregation is better described in physical terms rather than biological terms that imply preservation and transfer of biological information via replication and strain diversity. Amyloid aggregation is a process of nucleation-dependent phase transformation that is very common in nature similar to crystal growth or snow formation in non-biological systems. Moreover, other normal biological processes such as biomineralization of hard tissues (Veis and Dorvee, 2013) and the assembly of actin or tubulin (Job et al., 2003; Firat-Karalar and Welch, 2011) are also dominated by nucleation-dependent mechanisms. While many of these non-organic and organic phenomena share similar features with amyloids such as self-assembly, repeated patterns and superstructural polymorphism, in none of these cases is the process referred to as “self-replication” in the biological sense of the word. Moreover, polymorphic heterogeneity is dependent on the nucleation mechanism and environmental factors (such as pH, concentration, and temperature); factors that are not encoded in the core molecular conformation; and hence, cannot be faithfully replicated. Importantly, nucleation reactions take place via HEN, where no information is transferred from the catalyzing surface to the growing fiber, while still affecting superstructural polymorphism. This lack of information preservation or transfer indicates that the amyloid/prion phenomenon cannot be compared to the nucleic acids in terms of biological replication; which in the case of nucleic acids, is dominated by well-controlled mechanisms and machinery that ensure preservation and faithful replication of the genetic information (Table 1). In that sense, amyloid/prion heterogeneity cannot also be compared to biological strains in terms of the fidelity of storage and transfer of biological information, and thus, we opt for the term polymorphs instead, which is accordance with nomenclature for similar phenomena, such as crystalline polymorphs for example.

**TABLE 1:** The differences between the double helical structural architecture of DNA and the cross-β sheet architecture of amyloids in terms of the capability of both architectures to hold and transmit biological information.

Double helix	Cross-β sheet
Helical	β-sheet
Hydrated	Dry core steric zipper
Soluble	Insoluble
Pairing mechanism (A:T, C:G), ensures replication with fidelity	No specific pairing mechanism
Linear triplet code	No code
Specific sequence	Generic fibrillar structure
Open (major and minor grooves for protein binding)	Closed (no specific protein binding)
Can unwind	Cannot unwind
Dedicated machinery for unwinding, replication, and transcription	None
Low sensitivity to extrinsic factors (concentration, temperature, pH, and surface catalysts)	High sensitivity to extrinsic factors (concentration, temperature, pH, and surface catalysts)
Nucleation independent (active process that requires ATP)	Nucleation dependent (passive process dependent on the total free-energy of the system)
Organized in well-defined chromatin architecture, up to the level of chromosomes	Stochastic protofilament stacking depending on microenvironmental conditions

We are aware that the “protein-only” hypothesis of protein “self-replication” was initially introduced to distinguish amyloids from viral infections based on the absence of nucleic acids within amyloids (Zabel and Reid, 2015). Despite the historical importance of such distinction, it does not imply that the amyloid phenomenon should always be understood within the bounds of this historical dichotomy. Structural and biophysical studies of amyloids in recent years have uncovered important details about the common structural features of amyloids and the different physical pathways of their aggregation, as discussed above. Many of these new advances do not fit easily within the “protein-only” paradigm. This is particularly apparent in relation to HEN phenomena, which can be mediated by viral and other microbial surfaces; the very species the “protein-only” hypothesis was supposed to exclude from the pathology. HEN also clearly demonstrates the lack of information transfer during the amyloid aggregation process. That is why a new synthesis of the available data was necessary to accommodate for these findings. In that sense, we think that the nucleation-based classification of amyloid pathologies that we describe here does offer a more accurate and inclusive way to describe the multifactorial nature of amyloid aggregation using a well-defined physical framework.

One advantage of this physical classification is that it provides a mechanistic explanation of phenomena that are currently unaccounted for within the “protein-only” paradigm, including the sporadic amyloidopathies. It allows the integration of risk factors (such as lipid pathology, infections, and pollution) into the core of the pathogenesis via a well-defined mechanism; HEN. Furthermore, by highlighting the common physical foundations of the amyloid aggregation process, it becomes much easier to find correlations and common mechanisms between different amyloid pathologies that have been studied separately in isolated disease contexts. This creates a logical framework where data from different diseases can be integrated into a more general understanding. One outcome of such general understanding is that HEN and LOF mechanisms assume a more clear and prominent role in disease etiology and pathophysiology, opening new opportunities for novel diagnostic and therapeutic modalities. This is particularly important at a time where the failure of previous therapeutic interventions calls for new ways to understand amyloid pathologies.

In relation to novel diagnostics, HEN pathways are expected to contribute to amyloid polymorphism (see above), which can help in the differential histopathological diagnosis by relating particular amyloid polymorphs to certain HEN interactions. This may enable the development of new therapeutic interventions to specifically target these interactions, or preventive measures such as vaccines targeting specific microbes involved in HEN-mediated amyloid induction. In addition, highlighting the LOF angle of the pathology can lead to new treatments that aim to restore the original protein functions via different replacement therapy approaches.

## Conclusion: *Pathological Protein Polymerization*

While a number of proteins polymerize into filaments for functional purposes (actin and collagen for example), most proteins perform their functions in a soluble state. However, under certain circumstances, soluble proteins are pathologically nucleated to form fibrillar amyloid polymers. This uncontrolled nucleation leads to a conformational transformation from native conformation into the cross-β conformation and a phase transformation into solid fibrils. Such biochemical and biophysical transformations would lead to loss of biological function even if the resulting aggregates are not particularly more toxic. Within this framework, different etiologies of amyloid diseases can be linked to different mechanisms of nucleation. We point out that familial mutations facilitate spontaneous nucleation, leading to HON dominated mechanisms. The sporadic forms on the other hand may rely more on catalytic nucleation mechanisms, either via prion seeding or HEN. HEN mechanisms, in turn, can be mediated by a plethora of PNSs, among which microbial membranes such as viruses and bacteria may be of critical importance due to their ability to invade and replicate in various tissues. Furthermore, we propose that amyloids are more precisely described in physical terms similar to other organic and non-organic phase transformations, rather than in biological terms that invoke self-replication and biological strains. Such a generalized framework for a mechanistic-based understanding can open new avenues for the exploration of new measures to diagnose, prevent, and treat amyloidopathies.

## Author Contributions

KE conceived the concept and wrote the manuscript. MM, TM, OG, AS, CG, AE, AW, SE, and AL read and critically contributed to the manuscript.

## Conflict of Interest

The authors declare that the research was conducted in the absence of any commercial or financial relationships that could be construed as a potential conflict of interest.
